# A Study on the Design Depth of Permeable Road Pavement through Dynamic Load Experiment

**DOI:** 10.3390/ma15134391

**Published:** 2022-06-21

**Authors:** Chun-Hua Hsing, Jun-Han Siao, Yu-Min Wang

**Affiliations:** 1 Department of Civil Engineering, National Pingtung University of Science and Technology, Pingtung 91201, Taiwan; hsingchun0929025722@yahoo.com.tw (C.-H.H.); p10833002@g4e.npust.edu.tw (J.-H.S.); 2 General Research Service Center, National Pingtung University of Science and Technology, Pingtung 91201, Taiwan

**Keywords:** permeable road pavement, dynamic load experiment, mechanical properties, stress and strain, design depth

## Abstract

This study investigated vertical strain and stress through a dynamic load experiment at the testing area of Ke-Da Road, Pingtung, Taiwan. A thirty-five-ton truck was moved at constant speeds of 40, 60, and 80 km/h to simulate heavy load conditions to study the mechanical variations. From the results, it was found that the strain and stress curves of the permeable road pavement showed asymmetry due to the viscoelastic property of the open-grade friction course. The results showed that vertical strains and vertical stresses of permeable road pavement were greatly affected by the axle configuration and the change in traffic speed. Furthermore, to propose the design thickness of a permeable road pavement, the pavement strain and stress were modelled with respect to depth using regression based on these collected data. According to the stress regression models and considering the construction uncertainty, the recommend design depth of a permeable pavement is 30 cm. The findings of this study would be helpful in determining the permeable road pavement depth when subjected to heavy traffic load, and the material combination of open-graded friction concrete, porous asphalt concrete, and permeable cement concrete was proposed in this study during the design period.

## 1. Introduction

Due to the connected pores, the strength and the life-cycle of permeable road pavements are mostly lower than those of conventional pavement under repeated moving loads of traffic [1,2,3,4]. Under this dynamic load condition, the pavement can face failure from serious rutting and fatigue cracking. Thus, the thickness of the permeable road pavement design must be sized to support the moving traffic loads to ensure a longer life. In the past, permeable road pavements were mostly applied in light-traffic areas rather than heavy-traffic areas [3,5,6]. In other words, the application scope of permeable road pavements is limited [4,6].

Many researchers have investigated pavements’ mechanical responses with several methods, such as field tests [7,8,9,10,11,12,13,14], theoretical analysis [15,16,17], and finite-element method simulations [4,9,14,15,16,17,18,19,20,21,22,23,24,25,26]. Firstly, some research has studied the pavement responses through field testing. In El-Hakim’s study [7], the tensile strain in asphalt layers, vertical pressure on the subgrade surface, moisture in the subgrade material, and the temperature profile of three different pavements were monitored by researchers to evaluate the performance of the pavements. The three different pavements were perpetual pavement design with rich bottom mix (RBM) layer, perpetual pavement design without RBM, and a conventional pavement design. The results show that two perpetual pavements are subjected to lower stresses and corresponding strain values in the lowest and highest asphalt layers compared to the conventional pavement under the same traffic loads. Perpetual pavements have high structural resistance to different fatigue stresses due to their thick asphalt layers. These results reflect the better structural performance of the perpetual pavements in the long term. Sebaaly et al. [27] conducted comprehensive field testing program on strains, stresses, and displacements of asphalt concrete pavement induced by moving truck loads. The test results found that the vehicle speed has a significant effect on the tensile strains of asphalt concrete (AC). Moreover, it also found that the presence of a substantial compressive-strain at the bottom of the AC is important to evaluate the fatigue-life of the pavement. The maximum tensile strain, maximum compressive strain, and maximum compressive stress occurred below the tandem axle of the truck, especially the rear tandem axle. Al-Qadi et al. [8] also found that horizontal strains of the flexible pavement were strongly influenced by traffic speeds. Although the traffic speeds do not affect the magnitude of the vertical compressive stress, the speed does affect the loading time. In addition, it was found that the hot mixture asphalt (HMA) pavement had high horizontal strains during compaction when used by a steel drum compactor with vibrations. Chen et al. [10] conducted a series of full-scale field tests to observe the dynamic responses of the composite pavement, which is composed of an HMA surface, cement-treated bound base, and the red residual soil subgrade. The results show that the lower dynamic responses of the composite pavement occur at a higher truck speed due to the viscoelastic property of the HMA layer. A lower moving speed with a heavy weight would amplify the vibration of the pavement and result in larger dynamic responses in the composite pavement.

Secondly, after field testing, Zafir et al. [18] developed an efficient moving-load model to predict the strain, stress, and deflection, as well as the effect of the important dynamic from the moving loads, based on the previous work by Sebaaly et al. [27]. The modeling result showed that the strain is impacted by the thickness and vehicle speeds, and pavement strains substantially reduce with the increase in vehicle speed. Siddharthan et al. [19] presented the pavement responses of the finite-layer analytical model and 3D-Moving Load Analysis under a series of loading conditions. This reveals that the vehicle speed has a significant impact on the pavement response. The magnitude of the calculated pavement strain response decreased with the increase in vehicle speed. Ahmed et al. [20] developed a dynamic finite element model (FEM) of an instrumented pavement section on Interstate-40 near Albuquerque, New Mexico in ABAQUS, incorporating depth-temperature variations in the AC layer under truck wheel loading. Researchers found that the horizontal tensile strain decreases as the horizontal modulus of the AC increases. The increase in the horizontal modulus of the AC leads to decrease in the vertical strains of pavement layers. In addition, temperature variations in the AC layer are highly sensitive to horizontal tensile strains at the bottom of the AC layer and vertical strains on the top, base, subbase, and subgrade of the pavement. Although the pavement’s dynamic response is simulated by the FEM, the field pavement responses are still unclear due to the limitations of the testing conditions [10].

Generally, the tensile strains of the asphalt surface, measured by strain transducers in the pavement, were used to evaluate the bottom-up fatigue damage [9,28]. In the laboratory, the tensile and compressive strains of the pavement under loading pulses were also often used to determine the fatigue strength [18]. Thus, it is necessary to measure the vertical strains in the permeable road pavement to determine the pavement damage. Moreover, the vertical pavement stress is related to permanent deformations in the base and subgrade of the pavement. It is easy produce large settlements and rutting in the pavement when the pavement stress is high [11]. For instance, previous research showed that the huge increase in vertical stress in the unbounded granular base was presented before and after the destruction of the polyurethane-bound permeable material surface [12]. Hence, it is necessary to investigate the strain and stress responses in the permeable road pavement.

Pavement responses under the dynamic loads were affected by the material properties, the pavement thickness, the bonding condition between pavement layers, vehicle speeds, and the axle load amplitude [29,30]. Although some studies have reported on the mechanical responses of asphalt pavement, few of the literature have documented the mechanical responses of permeable road pavements. To understand and expand on the applications of permeable pavement, the vertical strains and stresses of permeable road pavement under heavy vehicle loadings with three different designed speeds were investigated and studied without consideration of temperature, pore water pressure, and moisture.

## 2. Materials and Methods

A series of dynamic load experiments were conducted in this study to investigate the permeable road pavement responses. The dynamic load experiments were conducted in the study area of the permeable road pavements in Ke-Da road, Pingtung, Taiwan. A 35-ton truck was used in this study and moved at constant speeds of 40, 60, and 80 km/h to model heavy load conditions.

### 2.1. Study Area

The study area (N 22°39′44.6268″, E 120°33′38.376″) is located at Section III of Ke-Da Road, between 15k + 125 and 15k + 225, route 187 of Pingtung County, Taiwan. Ke-Da Road is a dual carriageway with a 3-m rapid-vehicle lane and a 5.5-m mixed-vehicle lane, in both north and south driving directions. This road is the main route between National Pingtung University of Science and Technology and Pingtung City. The permeable road pavement test area of Ke-Da Road was constructed in June 2018. The pavement consists of three layers: 3 cm-thick open-graded friction concrete (OGFC), 10 cm-thick porous asphalt concrete (PAC) at surface course, and 40 cm-thick permeable cement concrete (PC) at base course.

Air void content is the basic property of permeable road pavement. The OGFC content was 16.4% by volume. Due to the high void content of the OGFC, water can quickly be removed and a rough surface structure can be formed. The OGFC has good skid resistance and high permeability. With the same benefits, the air void content of PAC was 17.1% in volume under the wearing course. The maximum nominal aggregate size of OGFC was 3/8″ (9.5 mm). However, the surface course used PAC mixed with the modified Type III asphalt, with a maximum nominal aggregate size of 3/4″. The gradations of each material are presented in Table 1. The basic physical properties of surface course materials are listed in Table 2. The applications of permeable PC have great potential environmental benefits. The stormy rainfall can be well-managed and the groundwater quality can be improved via the water infiltration process. At a 0.44 water–cement ratio, the proportion of PC on the site was 261 kg cement, 135 kg water, 980 kg aggregate of 3/4″, 653 kg aggregate of 3/8″. The average compressive strength after 7 and 28 days of PC was 112.8 and 142.2 kg/cm^2^, respectively, as shown in Table 3. However, voids in the PAC and PC pavement were easily clogged within three years after construction due to sediment and dust, etc. [31].

### 2.2. Monitoring Instruments

**Figure 1:**
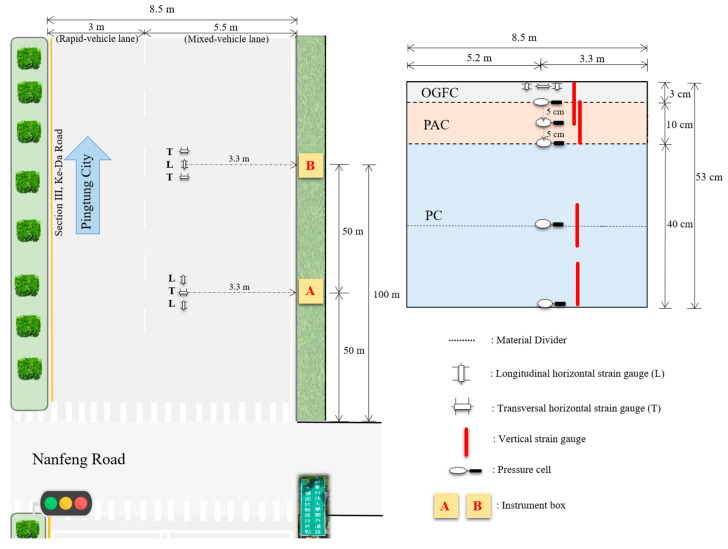
Top and profile view of pavement instruments (not to scale).

The layout of the instruments used in this study is shown in Figure 1. Each layer was equipped with eight vertical strain transducers (KM-100 B), also from Tokyo Sokki Kenkyujo Co., Ltd. (Tokyo, Japan) to measure the dynamic strains. Ten earth pressure cells (Model 3500 Series) from Geokon, Inc. (Lebanon, NH, USA) were installed to measure the vertical stress at the PAC of upper, middle, and lower layers. All gauges were installed in the mixed-vehicle lane and in the right wheel path, and evenly distributed between the two areas (area A and area B). During the tests, two data acquisition systems DSPL-24 from Geomaster were used to acquire the data from strain transducers and pressure cells in the permeable road pavement. DSPL-24 can measure the velocity and acceleration at a high resolution. The sampling rate of DSPL-24 was 50 samples per second.

Equations (1) and (2) are used to calculate the strains and the applied pressures in the field tests.

(1) $$\varepsilon_{1}=C_{\varepsilon }\times \varepsilon_{i}.\;$$

where $\varepsilon_{1}$ is the strain, ×10^−6^; $C_{\varepsilon }$ is the calibration coefficient, ×10^−6^/1 × 10^−6^, as shown in Table 4; $\varepsilon_{i}$. is the change in measured value from the initial value (×10^−6^).

(2) $$P=\left(R_{1}-R_{0}\right)\;G.\;$$

where P is the applied pressure, kPa; $R_{1}$. and $R_{0}$. are the current and initial output reading in millivolts, volts or milliamps; G is the gage factor, as shown in Table 5. (Note that gage factor is for an excitation of 10 volts. For any other excitation voltage, V, the gage factor shown must be multiplied by 10/V.)

### 2.3. Dynamic Load Experiment

**Figure 2:**
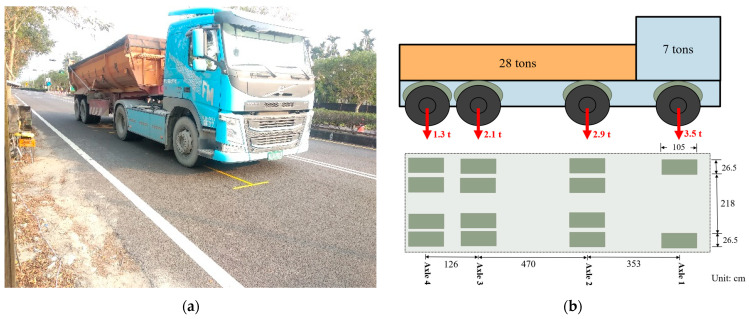
(**a**) A 35-ton truck from the Seven Seas Transportation Company; (**b**) configuration and dimensions of wheel axle.

The dynamic load experiment was carried out on 25 October 2019. During the experiment, the average atmosphere temperature was 27.5 °C, and there was no rainfall. The 35-ton semitrailer truck from the Seven Seas Company (see Figure 2a) was used. The weight of the semitrailer truck was determined according to the limited total weight of the semitrailer of Road Traffic Safety Rules of the Ministry of Justice of the Republic of China (Taiwan) [32]. Figure 2b shows the configuration of the truck axles, and individual static wheel load was calculated using the lumped mass method. There were fourteen 315/80 R22.5 wheels in the experiment truck. The static loads for each wheel and the standard pressure of the wheels are listed in Table 6.

In addition, the limited speed of vehicles on Ke-Da road is 60 km/h, according to the Road Traffic Safety Rules. The 40 km/h and 80 km/h truck speeds also were performed to study the difference between strain and stress responses. The truck moved along the pr-marked path for the right wheel in the mixed-vehicle lane and with constant speeds of 40, 60, and 80 km/h during tests. A video recorder was placed on the field to ensure the truck wheels’ passage on the positions.

### 2.4. Data Process

Through dynamic load experiments, the data of various depths of permeable pavement were obtained. During the experiment, it was difficult for the outer wheels of the truck to accurately press the sensor position, and hard to produce meaningful strain and stress data in every trial. Therefore, it is necessary to process data after field experiments. The acceptable raw data were extracted through the following procedures.

1. Four peaks in the strain and stress of the permeable road pavement must be detected by the instrument. The four peaks indicate that the wheel pressed against the instrument;
2. The detected values have to have positive pressure and positive tension;
3. The stress and strain responses of the surface layer have to be larger than those of the base layer;
4. The faster the vehicle speed, the smaller the stress and strain responses in the pavement, which is consistent with the literature [10,19,27];
5. The time difference between the two truck axles should be calculated to determine if the trial is acceptable for the consideration of speed.

### 2.5. Relationship between Permeable Road Pavement Strain and Stress with Respect to Pavement Depths

Based on attenuation pattern results at 40, 60, and 80 km/h, a logarithmic regression analysis was conducted with the regression reference methods used in previous studies [33,34,35,36]. The peak values of maximum vertical strains and vertical stresses caused by the fourth wheel were selected and plotted on the logarithmic regression. A logarithmic regression analysis was performed on the plot, showing the maximum vertical strains and vertical stresses versus the depth. The following equations were derived, as shown in Equations (3) and (4). To avoid obtaining negative values for the logarithmic plot, 0 cm depths were omitted and the regression analysis was performed from 1 cm depths onwards.

(3) $$y_{vs,speed}=\alpha_{vs}x-\beta_{vs}\ln(x)+\gamma_{vs}$$

(4) $$y_{vp,speed}=\alpha_{vp}x-\beta_{vp}\ln(x)+\gamma_{vp}$$

where $\;{\;y}_{vs,speed}\;\;$ is the peak vertical strain of pavement for the specific speed of the truck; $y_{vs,\;speed}$ is the peak vertical stress of pavement for the specific speed of the truck, kPa; x is the specified pavement depth cm; $\alpha_{vs}\;$ and $\alpha_{vp}\;$ is the coefficient of the depth; $\beta_{vs}\;\;$ is the regression slope of the $y_{vs,speed}$−ln(x) plot; $\beta_{vp}$ is the regression slope of the $y_{vp,speed}$−ln(x) plot; $\gamma_{vs}$ is the regression intercept of the $y_{vs,speed}$−ln(x) plot; $\gamma_{vp}$ is the regression intercept of the $y_{vp,speed}$−ln(x) plot.

Based on the results of the Analysis of Variance (ANOVA) in the regression models, a series of tests were performed. Firstly, the test to determine the overall model adequacy was conducted using the *F*-test. If the model is deemed adequate, the β. coefficients in models were inferred using *t*-test.

## 3. Results and Discussion

### 3.1. Dynamic Responses of Permeable Road Pavement

#### 3.1.1. Vertical Strain

**Figure 3:**
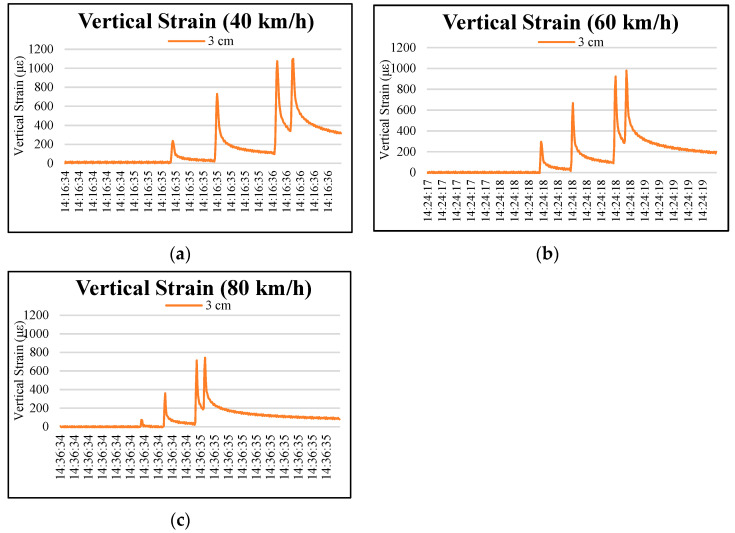
Typical vertical strain distributions at the depth of 3 cm in the permeable road pavement under different speeds: (**a**) truck speed of 40 km/h, (**b**) truck speed of 60 km/h, and (**c**) truck speed of 80 km/h.

**Figure 4:**
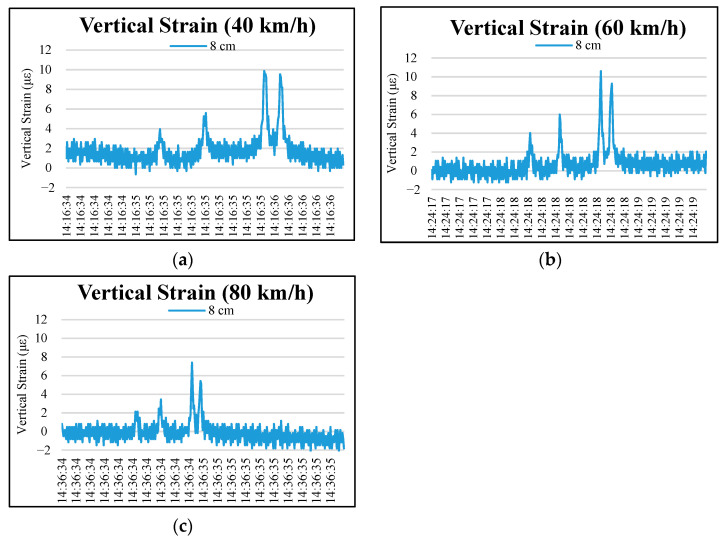
Typical vertical strain distributions at a depth of 8 cm in the permeable road pavement under different speeds: (**a**) truck speed of 40 km/h, (**b**) truck speed of 60 km/h, and (**c**) truck speed of 80 km/h.

Figure 3 and Figure 4 show the distributions of vertical strains at depths of 3 and 8 cm on the permeable road pavement under wheel loads at 40, 60, and 80 km/h truck speeds. As observed in the figures, the stresses and strains did not return to 0 after the truck wheel moved away the instrumented position, and the single-curve response shows asymmetry due to the viscoelastic property of the OGFC. The surface layer (3 cm and 8 cm) of the permeable road pavement is subjected to obvious tensile vertical strains under moving loads. Four peaks occurred at 3 cm and 8 cm pavement depths. The vertical strains caused by the third and fourth wheel axles dominate in the loading period at each truck speed.

Table 7 shows that, under the truck speeds of 40, 60 and 80 km/h, the maximum vertical strains of 3 cm depth in permeable pavement at the fourth peak were 1099.85, 979.46 and 742.13, respectively. The fourth peak vertical strain at the 8 cm depth was 9.54, 9.29 and 5.42, respectively, under 40, 60 and 80 km/h. Compared with the depth of 3 cm, the vertical strain value of 8 cm in the permeable road pavement was much lower. In addition, the increase in truck speed caused a vertical strain at the 3 cm pavement depth that was greater than that with the 8 cm pavement depth. The results show that increasing the speed has more influence on the vertical strain of the 3 cm pavement depth, as presented in the previous research [9].

**Figure 5:**
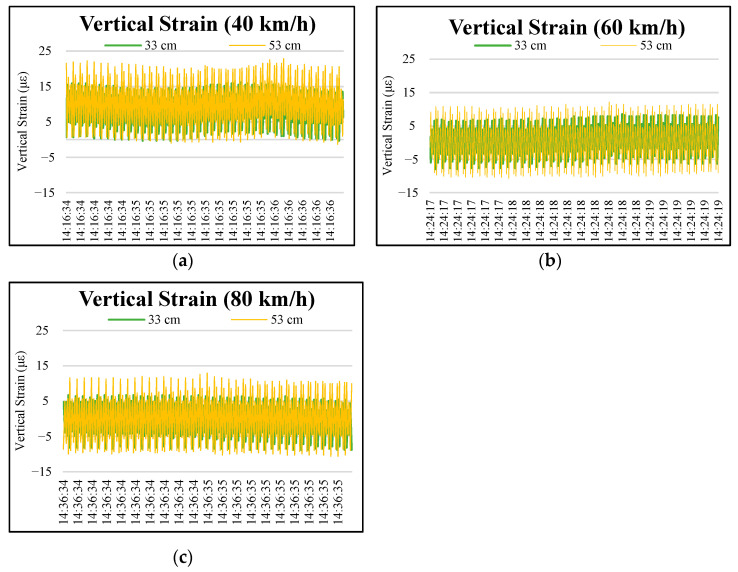
Typical vertical strain distributions at depths of 13 cm and 33 cm in the permeable road pavement under different speeds: (**a**) truck speed of 40 km/h, (**b**) truck speed of 60 km/h, and (**c**) truck speed of 80 km/h.

According to Lu et al. [26], with the increase in truck speed, the reaction time of a certain point on the road surface is reduced, thus reducing the strain on the road. The results of practical observations indicate that it is possible to extend permeable road pavement service life at higher traffic speeds over the long term. Moreover, the axle configuration and the truck speed have no effect on the vertical strain at the depths of 33 cm and 53 cm in the permeable road pavement, as shown in Figure 5.

#### 3.1.2. Vertical Stress

**Figure 6:**
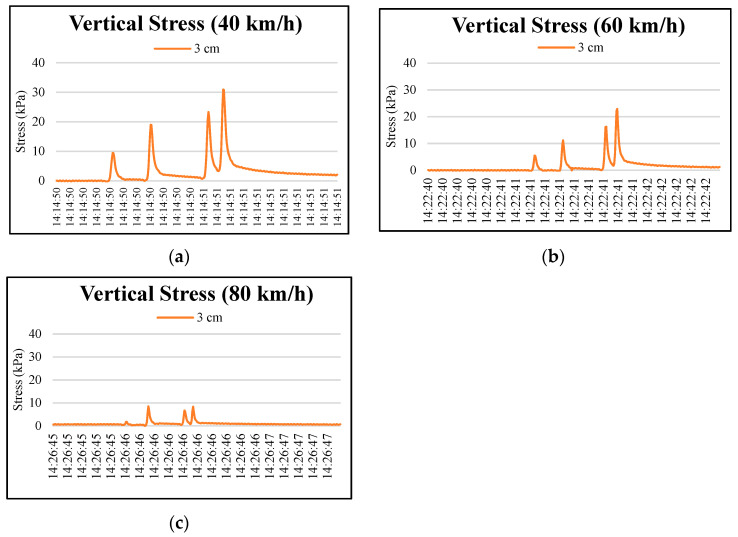
Typical vertical stress distributions at 3 cm depth in the permeable road pavement: (**a**) 40 km/h truck speed, (**b**) 60 km/h truck speed, and (**c**) 80 km/h truck speed.

**Figure 7:**
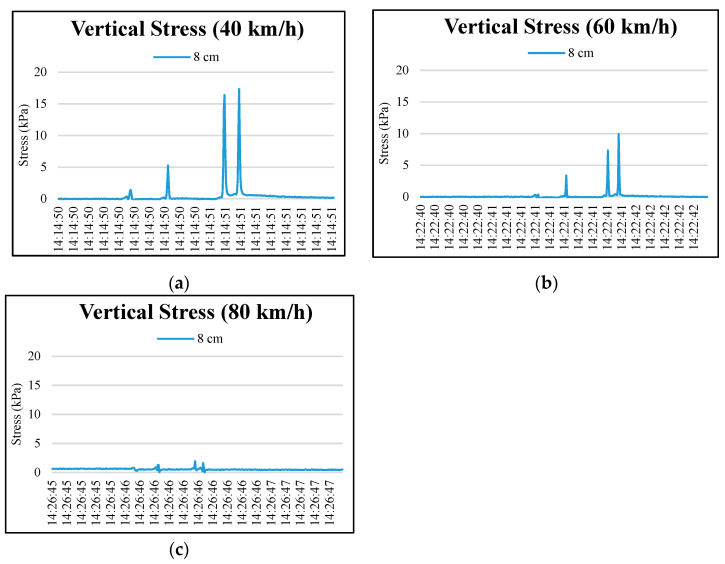
Typical vertical stress distributions at 8 cm depth in the permeable road pavement: (**a**) 40 km/h truck speed, (**b**) 60 km/h truck speed, and (**c**) 80 km/h truck speed.

**Figure 8:**
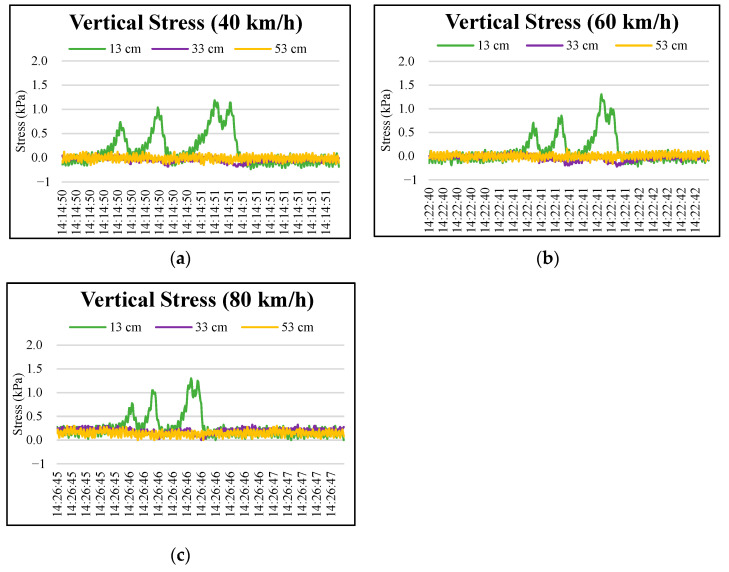
Typical vertical stress distributions at the depths of 13, 33, and 53 cm in permeable road pavement: (**a**) 40 km/h truck speed, (**b**) 60 km/h truck speed, and (**c**) 80 km/h truck speed.

Figure 6 and Figure 7 show four peaks in stress responses at 3 cm, 8 cm, and 13 cm pavement depths as the wheel axles passed pressure cells. The maximum stress response was recorded at the rear wheel axle of the truck. An accumulating stress effect was found from the third to the fourth peaks. This indicated that the axle configuration of the truck significantly influenced the vertical stresses of the upper layer of permeable road pavement. As expected, the lower layers of the permeable road pavement were less influenced by the wheel axles of the truck, as shown in Figure 8. The maximum values of stress responses at each depth are summarized in Table 8 to compare the dynamic stresses and traffic speeds. The results showed that maximum vertical stress values occurred at 3 cm and 8 cm depths at a 40 km/h test round. Conversely, the maximum vertical stress values at 3 cm and 8 cm depths at an 80 km/h traffic speed are the lowest of all of the truck speeds. For example, maximum vertical stress response values for the fourth peak at a 3 cm pavement depth were 30.76, 22.9, and 8.36 kPa, respectively, under traffic speeds of 40, 60, and 80 km/h. At an 8 cm depth, the vertical stress responses for the fourth peak were lowered to 17.34, 9.93, and 1.63 kPa, respectively, under traffic speeds of 40, 60, and 80 km/h.

### 3.2. Relationship of Permeable Road Pavement Strain and Stress with Respect to Pavement Depths

**Figure 9:**
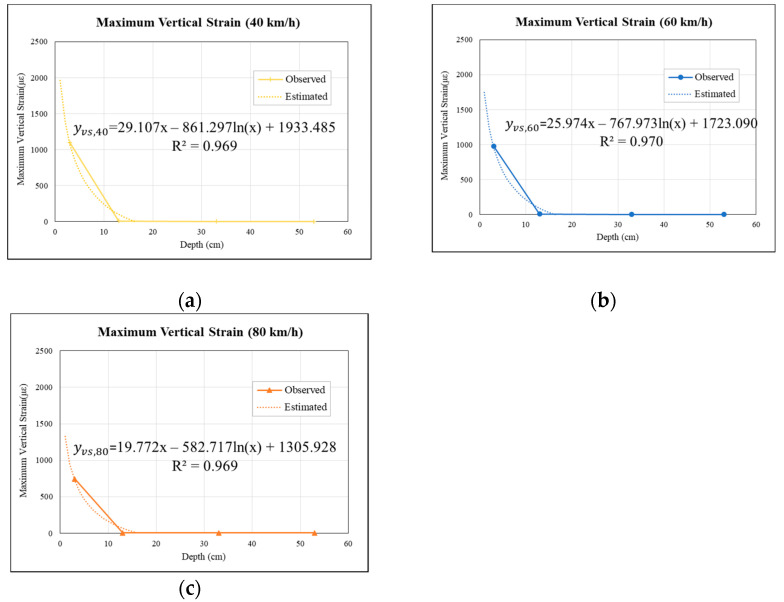
Relationships between vertical strains and permeable road pavement depths: (**a**) model for 40 km/h, (**b**) model for 60 km/h, and (**c**) model for 80 km/h.

The aforementioned results were used to evaluate the relationship between maximum vertical strains and stress with respect to pavement depth, as shown in Figure 9. The trends show that the maximum vertical strain decreases while the depth increases. The figure clearly shows that the response at the 3 cm pavement depth is the largest, and the response rapidly decreases at the 8 cm depth; as the depth increases, the strain response approaches zero.

Meanwhile, multiple regression models were developed to estimate vertical strains at different pavement depths, as presented in Figure 9. These models all performed well, with R^2^ coefficients of 0.97. Table 9 shows the results of an ANOVA F test analysis for the vertical strain models. The F value for each model was compared to the critical F value (F_crit_). The F values were 15.815, 16.345, and 15.772 for models of 40, 60, and 80 km/h, respectively. These F values are lower than the F_crit_ (18.513), indicating that H_0_ is acceptable. The effect of truck-driving speed on the depth in terms of maximum vertical strains and maximum vertical stresses was insignificant.

**Figure 10:**
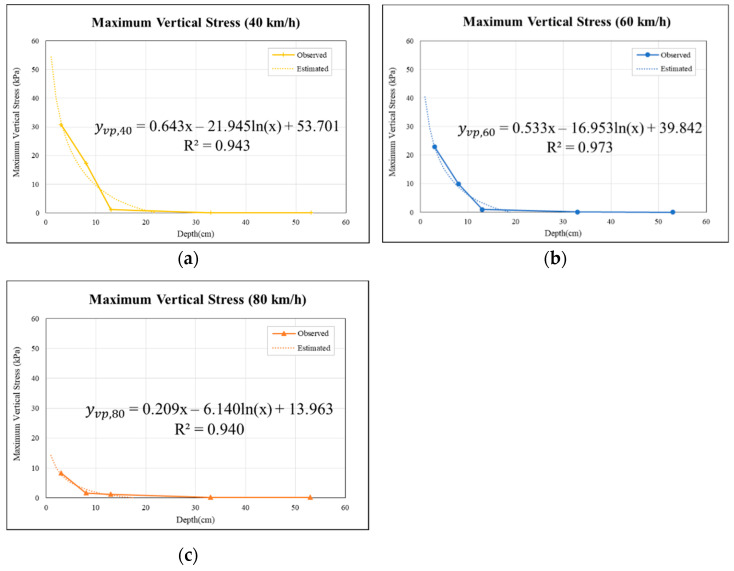
Relationships between vertical stresses and permeable road pavement depths: (**a**) model for 40 km/h, (**b**) model for 60 km/h, and (**c**) model for 80 km/h.

On the other hand, permeable road pavement stresses with respect to pavement depths at traffic speeds of 40, 60, and 80 km/h of traffic are shown in Figure 10a–c. It can be observed that the internal permeable road pavement stresses obviously decreased as depths increased. However, the influence under the lower traffic speeds for porous asphalt pavement (from 3 to 8 cm) was smaller than that under higher traffic speeds. Moreover, regardless of traffic speeds, the stresses were close to zero below the 30 cm depth layers. As shown in Figure 10, the R^2^ coefficients of regression for the models of 40, 60, and 80 km/h were 0.943, 0.973, and 0.940, respectively.

Table 10 shows the results of the ANOVA F test analysis for the vertical stress models at different speeds. The F values were 16.697, 35.853, and 15.742 for models of 40, 60, and 80 km/h, respectively. These F values were all higher than the F_crit_ (10.128), which means that H_0_ is rejected. Thus, the effect of pavement depth on maximum vertical stresses under different truck speeds was suggested to be significant.

After confirming that the multiple regression model is significant through the F test, a test of the individual coefficients in the multiple regression model was performed. The calculated *t*-values were 2.0315, 3.3775, and 2.5805 for the models of 40, 60, and 80 km/h, respectively, which are larger than *t*_0.1_ (1.886), as shown in Table 11. *p*-values for these models were larger than 0.05. Thus, the null hypothesis is rejected, indicating that the independent variables for the 40, 60, and 80 km/h models are meaningful.

Moreover, the regression formulas estimated that, when the vertical stress of the pavement is 0 KPa, the pavement depths will be 22, 19, and 18 cm at speeds of 40, 60, and 80 km/h, respectively. To widely apply the permeable road pavement in heavy traffic loading areas, the depths estimated by the stress regression model are used to suggest the design depth value of the permeable road pavement for heavy-load traffic designs. Considering the condition of pavement overload, it is necessary to introduce a specified safety factor in the design. Therefore, the theoretical results obtained through these models are multiplied by a safety factor of 1.2 to 1.5 according to the suggestion of highway-graded classification. Thus, the recommended design depth of the permeable road pavement is 30 cm, and this result is the same as the study of Yang et al. [37].

## 4. Conclusions

This paper presents the mechanical responses of the permeable road pavement and the design thickness of the pavement through the established models of depth-vs.-stress and depth-vs.-strain. The main findings of this field investigation and regression model led to the following conclusions:

- The result of vertical strains and vertical stresses in permeable road pavement indicated that the stresses and strains did not return to 0 after the truck wheel moved away from the instrumented position, and the single-curve response performs asymmetrically due to the viscoelastic property of the OGFC.
- The vertical strains and vertical stresses at the OGFC and the PAC showed an accumulating effect from the third to fourth axle from the collected data. Thus, the axle configuration significantly influences the strain and stress values of the permeable road pavement.
- Vertical strains and stresses of the permeable road pavement were strongly influenced by the truck driving speeds. An increase in truck speed decreases the reaction time of a point on the pavement, which reduces the pavement strain. This indicates that it is possible to extend the service life of a permeable road pavement at reasonably higher traffic speeds.
- By using the ANOVA F-test and *t*-test, from these models, the multiple logarithmic regression models of vertical stresses vs. vehicle speeds of 40, 60, and 80 km/h are meaningful. The estimated depths of the permeable road pavements are 22, 19, and 18 cm at speeds of 40, 60 and 80 km/h, respectively. Considering the results and the construction uncertainty, the recommended design thickness of the permeable pavement is 30 cm under the OGFC, PAC, and PC combination reported in Table 1 and Table 2, while the dynamic impacting force is not under consideration.

## Figures and Tables

**Table 1:** Gradations in surface and base materials.

Sieve No.	Opening (mm)	Percent Passing (%)		
		OGFC	PAC	PC
$1\frac{1}{2}$″	37.5	100	100	100.0
1″	25.0	100	100	100.0
3/4″	19.0	100	95	89.5
1/2″	12.5	100	72	50.0
3/8″	9.5	86	52	27.8
No. 4	4.750	38	18	1.6
No. 8	2.360	15	12	-
No. 10	2.000	8	9	-
No. 30	0.600	5	8	-
No. 50	0.300	4	6	-
No. 100	0.150	3	5	-
No. 200	0.075	2.0	3.6	-

Note. Sieve No. refers to the number of meshes in the area of one square inch, at which the particle can pass through the mesh.

**Table 2:** Basic physical properties of OGFC and PAC in the surface course.

Type	Thickness (m)	Asphalt Content (%)	Maximum Specific Gravity (25 °C/25 °C)	Maximum Density (Unit Weight) (kg/m^3^)	Porosity, N (%)	Permeability (cm/s)	Stability (N)	Retained Strength Index (%)
OGFC	0.03	4.5	1.021	1018	16.4	-	4446	93.5
PAC	0.10	4.9	2.442	2435	17.1	0.21	3329	80.2

**Table 3:** Basic properties of PC in base course.

Cement Adjust, C (kg)	Water Adjust, W (kg)	Amount of Aggregate, G (kg)		Total Material Amount, W_T_ (kg)	Average Compressive Strength (kg/cm^2^)		Permeability (cm/s)	Porosity, N (%)
		19.0 mm	9.5 mm		7 days	28 days		
261	135	980	653	2029	112.8	142.2	1.642	17.6

**Table 4:** Calibration coefficients of vertical strain transducers from the operation manual of the vertical strain transducer.

Area	Sensor	Sensor Location (cm)	Instrument Serial Number	Calibration Coefficient
A	Vertical strain transducer	3	EKD150270	0.804
		8	EKD180339	0.828
		33	EKD180338	0.822
		53	EKD150276	0.816
B	Vertical strain transducer	3	EKD180342	0.808
		8	EKD180342	0.822
		33	EKD180341	0.826
		53	EKD180340	0.829

**Table 5:** Gage factors of earth pressure cells from the operation manual of the earth pressure cell.

Area	Sensor Location (cm)	Instrument Serial Number	Gage Factor
A	3	1743901	25.034
	8	1743902	25.041
	13	1743903	25.053
	33	1743904	25.010
	53	1743905	25.030
B	3	1743906	25.046
	8	1743907	24.658
	13	1743908	25.009
	33	1743909	25.009
	53	1743910	25.027

**Table 6:** Static loads for the experiment truck weight and tire pressure.

Type of Axle	Truck-Axle Configuration	Number of Wheels	Weight of Outer Wheel (ton)	Weight of Inner Wheel (ton)	Tire Pressure (MPa)
Single	Axle 1	2	3.5	-	0.85
Single	Axle 2	4	2.9	3.5	
Tandem	Axle 3	4	2.1	2.6	
	Axle 4	4	1.3	1.6	

**Table 7:** Maximum vertical strains of each axle at each depth in the permeable road pavement at different speeds.

Maximum Values of Vertical Strains (*με*)													
Speed		At 40 km/h				At 60 km/h				At 80 km/h			
	Peak	First	Second	Third	Fourth	First	Second	Third	Fourth	First	Second	Third	Fourth
Depth (cm)													
3		235.29	729.79	1074.96	1099.85	295.89	666.60	922.90	979.46	73.11	359.14	713.37	742.13
8		3.95	5.59	9.86	9.54	4.03	6.00	10.60	9.29	2.13	3.45	7.39	5.42
33		3.97	1.98	5.95	5.62	1.61	2.27	5.24	2.93	6.04	6.04	6.04	4.06
53		0.00	1.33	2.98	1.33	1.56	1.56	2.88	2.22	1.32	1.65	2.64	2.97

**Table 8:** Vertical stresses at each permeable road pavement depth under different speeds.

Maximum Values of Vertical Stress (KPa)													
Speed		At 40 km/h				At 60 km/h				At 80 km/h			
	Peak	First	Second	Third	Fourth	First	Second	Third	Fourth	First	Second	Third	Fourth
Depth (cm)													
3		9.39	18.96	23.32	30.76	5.39	11.17	16.13	22.91	1.75	8.49	6.71	8.36
8		1.42	5.28	16.39	17.34	0.37	3.37	7.35	9.93	0.83	1.3	1.93	1.63
13		0.74	1.04	1.19	1.14	0.71	0.86	1.31	1.01	0.78	1.05	1.3	1.25
33		0.07	0.05	0.07	0.07	0.06	0.06	0.08	0.08	0.18	0.23	0.18	0.00
53		0.04	0.00	0.04	0.04	0.05	0.00	0.03	0.00	0.08	0.00	0.03	0.18

**Table 9:** Results of ANOVA F test of vertical strain models at different speeds.

Models for Specific Speeds (km/h)	Source of Variation	Df	Sum of Squares	Mean Squares	F	Sig.	F_crit_ (α = 0.05)
40	Regression	2	870,716.304	435,358.152	15.815	0.175	18.513
	Residuals	1	27,528.198	27,528.198	―	―	
	Total	3	898,244.501	―	―	―	
60	Regression	2	691,336.794	345,668.397	16.345	0.172	18.513
	Residuals	1	21,148.071	21,148.071	―	―	
	Total	3	712,484.865	―	―	―	
80	Regression	2	395,911.518	197,955.759	15.772	0.175	18.513
	Residuals	1	12,551.242	12,551.242	―	―	
	Total	3	408,462.760	―	―	―	

**Table 10:** Results of ANOVA F test of vertical stress models for different speeds.

Model for Specific Speed (km/h)	Source of Variation	Df	Sum of Squares	Mean Squares	F	Sig.	F_crit_ (α = 0.05)
40	Regression	2	717.958	358.979	16.697	0.057	10.128
	Residuals	2	42.999	21.500	―	―	
	Total	4	760.957	―	―	―	
60	Regression	2	383.609	191.804	35.853	0.027	10.128
	Residuals	2	10.700	5.350	―	―	
	Total	4	394.308	―	―	―	
80	Regression	2	44.179	22.089	15.742	0.060	10.128
	Residuals	2	2.806	1.403	―	―	
	Total	4	46.985	―	―	―	

**Table 11:** Results of ANOVA *t*-test of vertical stress models for different speeds.

Model for Specific Speed (km/h)	Source	Coefficient	Std.Error	*t*-Value	*p*-Value	Lower Bound (95%)	Upper Bound (95%)	*T*_0.1_
40	Intercept	53.7011	9.2729	5.7912	0.0285	13.8030	93.5992	1.886
	Depth	0.6431	0.3166	2.0315	0.1793	−0.7190	2.0053	
	ln(x)	−21.9454	5.7582	−3.8112	0.0625	−46.7209	2.8301	
60	Intercept	39.842	4.6256	8.6134	0.0132	19.9400	59.7448	1.886
	Depth	0.533	0.1579	3.3775	0.0776	−0.1461	1.2128	
	ln(x)	−16.953	2.8724	−5.9022	0.0275	−29.3120	−4.5945	
80	Intercept	13.9630	2.3690	5.8942	0.0276	3.7702	24.1559	1.886
	Depth	0.2087	0.0809	2.5805	0.1231	−0.1393	0.5567	
	ln(x)	−6.1399	1.4711	−4.1738	0.0529	−12.4694	0.1895	
